# Smooth Things Come in Threes: A Diabatic Surrogate Model for Conical Intersection Optimization

**DOI:** 10.1021/acs.jctc.3c00389

**Published:** 2023-05-16

**Authors:** Ignacio Fdez. Galván, Roland Lindh

**Affiliations:** † Department of Chemistry−BMC, Uppsala University, P.O. Box 576, SE-75123 Uppsala, Sweden; ‡ Uppsala Center for Computational Chemistry (UC_3_), Uppsala University, P.O. Box 576, SE-75123 Uppsala, Sweden

## Abstract

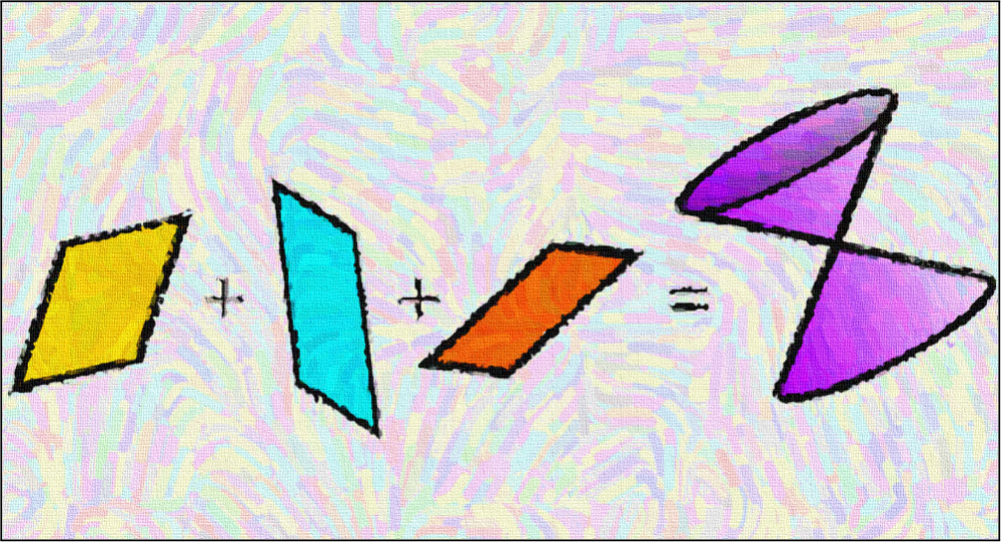

The optimization of conical intersection structures is complicated by the nondifferentiability of the adiabatic potential energy surfaces. In this work, we build a pseudodiabatic surrogate model, based on Gaussian process regression, formed by three smooth and differentiable surfaces that can adequately reproduce the adiabatic surfaces. Using this model with the restricted variance optimization method results in a notable decrease of the overall computational effort required to obtain minimum energy crossing points.

## 1 Introduction

Nonadiabatic processes, in which more than one Born–Oppenheimer potential energy surface (PES) affect the nuclear motion, are involved in many photophysical and photochemical phenomena, such as vision,^1^ chemi- and bioluminescence,^2^ DNA photostability,^3^ photosynthesis,^4^ etc. The theoretical study of such processes has been greatly developed during the past couple of decades and involves mostly the use of quantum or mixed quantum-classical molecular dynamics simulations.^5,6^

One of the characteristics of nonadiabatic processes is the degeneracy or near-degeneracy between adiabatic electronic states. A particular salient feature is the existence of conical intersections (CIs), which have also been the object of a multitude of recent works.^7−11^ The geometries or structures where CIs occur are not isolated but form a continuous subspace of geometries, and the most relevant regions of this subspace will be those most frequently traversed during the dynamics. It is common, however, to carry out static studies, as a complement or instead of dynamics simulations, where only the geometries with lowest energies are identified.^12−15^ These geometries are known as minimum energy CIs, or more generally as minimum energy crossing points (MECPs).

The location of significant points in PESs is a fundamental task in computational studies. Over the years, a conventional paradigm for geometry optimization has emerged as robust and efficient and is the most commonly used.^16^ This is based on a second-order Taylor expansion of the PES, a step size restriction, approximate Hessian and Hessian update methods. A prime example of such “conventional” methods is the restriced-step rational function optimization (RS-RFO) in redundant internal coordinates.^17^ The second-order expansion has some limitations, in particular it cannot accurately represent the parent surface beyond a local region around the expansion point, and this has pushed us to propose and develop an alternative optimization scheme based on a more flexible surrogate model. The new method, which we have called restricted variance optimization (RVO),^18−20^ relies on a surrogate model generated with a Gaussian process regression (GPR) variant also known as gradient-enhanced Kriging (GEK).^21−23^ The most relevant differences with respect to similar methods proposed by other authors^24−26^ is that RVO uses the empirical knowledge encoded in the approximate Hessian model function (HMF)^27^ to define the so-called characteristic lengths of the model in internal coordinates and that it uses the predicted uncertainty of the model to restrict the displacement during the iterations.

For the specific case of MECP optimization, there have been a number of proposed methods, generally using projection techniques or penalty functions to ensure that the energies of two crossing states are degenerate and simultaneously minimize their value.^28−34^ We have previously used the projected constrained optimization method (PCO)^31,35,36^ to successfully optimize MECPs by including a constraint involving the energy difference. However, adapting this method to RVO is not straightforward. First, although purely geometrical constraints have been implemented,^19^ including the energy difference would require a surrogate model that can represent accurately the energy difference itself and its gradient. Second, the very nature of a CI means that the PESs involved in the crossing are not differentiable at the crossing points, and this poses challenges for a surrogate model that relies on differentiability such as GEK. Lastly, for an efficient location of CIs, knowledge of the nonadiabatic coupling vector, or a sufficiently good approximation, is very valuable, and it would be desirable to include this in the surrogate model as well.

In this work, we extend the RVO method to allow optimization of MECPs, either between states of the same spacial and spin symmetry (CIs) or different symmetries (e.g., singlet–triplet crossings). To this end, we build a pseudodiabatic surrogate model from the data (energies, gradients, and couplings) of the previous iterations. The model consists of three separate smooth and differentiable surfaces (two in the case of different-symmetry crossings) that when combined can reproduce the energies, gradients, and couplings of the parent method and thus can be used in combination with the constrained RVO.^19^ In section 2, the methodological details relevant for this work are detailed, in particular the construction of a surrogate model consistent with the presence of CIs. The performance of this method was tested in a set of MECP optimizations, for which the computational details are given in section 3, and the corresponding results are discussed in section 4. Finally, we summarize the work in section 5.

## 2 Theory and Methods

This section includes a summary on the local description of conical intersections, followed by details on how to switch between diabatic and adiabatic representations, and how a smoothly interpolating surrogate model is constructed, to finish with a short overview of the optimization method.

### 2.1 Conical Intersections

Conical intersections are features of most molecular systems, where two adiabatic electronic PESs are exactly degenerate (although not all degeneracies correspond to CIs). They were once considered an academic curiosity, but they are nowadays known to be ubiquitous in molecular systems and with very significant conquences for their photophysical and photochemical behavior. CIs have been extensively studied and described before,^7−11,37−41^ and here only the most relevant aspects for the rest of the article will be given.

In the absence of spin–orbit coupling, the degeneracy at a CI is lifted linearly with the displacement when the geometry of the system is distorted in one of two independent directions (or any combination thereof), while it is maintained for any other orthogonal displacement. Thus, the set of geometries where the two surfaces touch, the *intersection space*, is a subspace of *K*–2 dimensions, where *K* is the dimensionality of the PES. At each point of the intersection space, the 2-dimensional subspace that breaks the degeneracy is known as the *branching plane*. The branching plane can be defined as the subspace spanned by two (generally nonorthogonal) vectors, the gradient difference, ***g***, and the nonadiabatic coupling (NAC), ***h***. For completeness, it is also useful to define the average gradient vector, ***s***. These can be obtained as
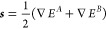
1
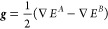
2
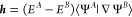
3where *E*^*A*^, *E*^*B*^ and Ψ^*A*^, Ψ^*B*^ are the energies and wave functions of the two degenerate states. In eq 3, the factor *E*^*A*^ – *E*^*B*^ (which is identically zero in the intersection space) effectively cancels an equal denominator in ⟨Ψ^*A*^|∇Ψ^*B*^⟩, such that a nonzero ***h*** is obtained.^36,37^ The degeneracy between the two states means that the two wave functions are not uniquely defined, as any unitary transformation of them is an equally good possibility. This also means that ***g*** and ***h*** are not uniquely defined for structures in the intersection space, but the branching plane—the subspace spanned by them—is. Indeed, a “rotation” of the two wave functions by an angle χ results in a corresponding rotation of the ***g*** and ***h*** vectors by an angle 2χ:
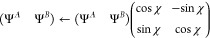
4
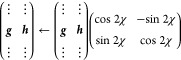
5where the vertical dots simply indicate that the vectors are arranged as columns. A pair of orthonormal vectors, ***x*^** and ***y*^**, unique up to permutations and sign flips (except in highly symmetrical cases), span the branching plane,^36^ and these are the coordinates used for the branching plane in the figures of this article.

**Figure 1:**
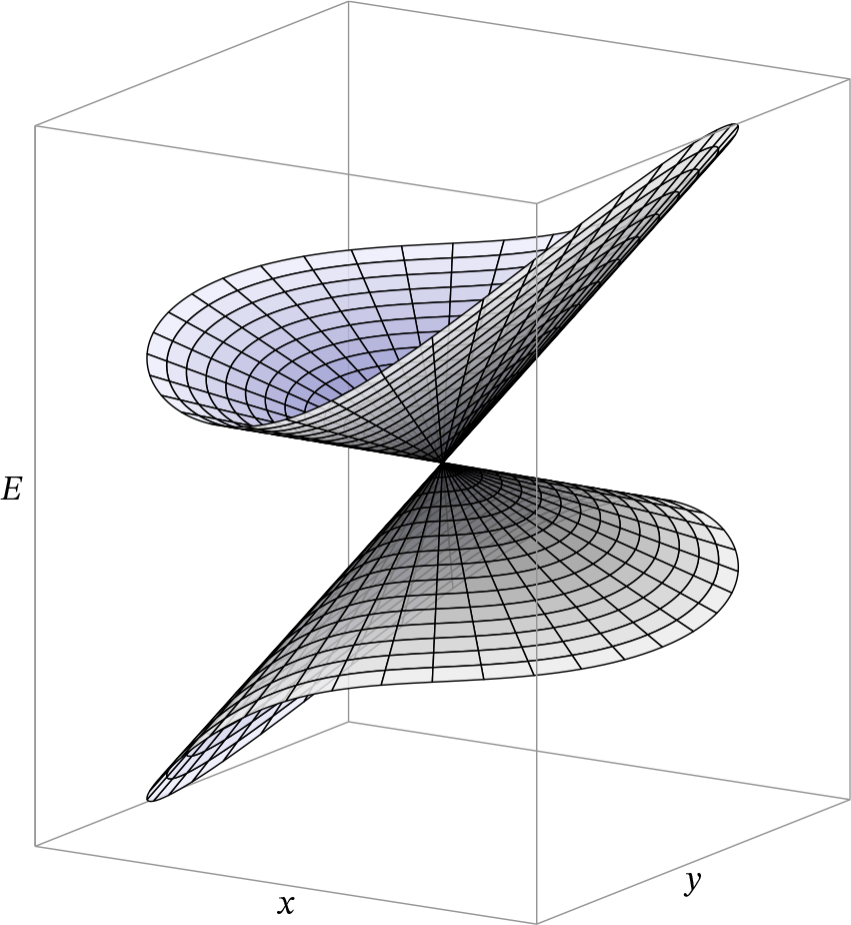
Representation of the adiabatic PESs around a conical intersection, in the branching plane, spanned by the *x* and *y* coordinates, with the energy along the vertical axis.

The adiabatic PESs around a CI, represented in the branching plane, have the familiar double-cone shape, with the two surfaces touching at the intersection point and diverging as the structure moves away from it (Figure 1). This shape makes the surfaces not differentiable at the intersection, which is problematic for optimization and dynamics methods, typically based on PES gradients. The location of CI structures, or in general of crossing points between adiabatic surfaces, is usually done by including some kind of constraint or penalty that enforces a zero energy difference between the surfaces.^28−30,32−34^ We make use of the PCO method,^31,35,36^ which allows general arbitrary constraints and requires, at each geometry, the adiabatic energies *E*^*A*^ and *E*^*B*^, and the ***s***, ***g***, and ***h*** vectors.

### 2.2 Diabatization

The lack of differentiability of the PESs can be avoided by switching to a diabatic representation of the electronic states, instead of an adiabatic representation. Such a transformation, known as diabatization, is commonly used in dynamics simulations, and there are many techniques to achieve it that are described and overviewed elsewhere.^42^ A strictly diabatic basis, in which the so-called nuclear-momentum coupling vanishes, does not in general exist.^43^ So, in practice, one resorts to a quasi-diabatic basis where the couplings are reduced to a negligible or acceptable size.^42^

In our case, our only goal is to obtain continuous, differentiable functions that can accurately reproduce the adiabatic surfaces around a CI. For this, we consider a simple linear two-state model, in which the elements of the Hamiltonian matrix are linear functions of the nuclear coordinates ***q***:^36,44,45^
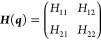
6
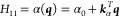
7
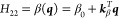
8
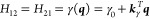
9Diagonalization of ***H*** yields the adiabatic energies *E*^*A*^ and *E*^*B*^ as eigenvalues. This can be compactly expressed in terms of the average (τ) and half-difference (δ) energies, and interpreting δ and γ as the two components of a vector, with modulus λ and argument ω:
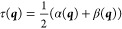
10
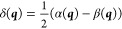
11
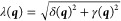
12
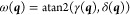
13where the function atan2(*y*, *x*) is similar to arctan (*y*/*x*) but returns an angle in the correct quadrant according to the signs of the two arguments. Then *E*^*A*^ and *E*^*B*^ are given by
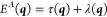
14
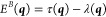
15The vectors ***s***, ***g***, and ***h*** are obtained from ***k***_α_, ***k***_β_, and ***k***_γ_:
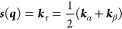
16
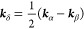
17
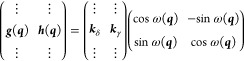
18We are interested in the reverse process (diabatization), i.e., obtaining the diabatic properties (α, β, γ) from the adiabatic ones (*E*^*A*^, *E*^*B*^, ***s***, ***g***, ***h***). This would be trivial if we knew the angle ω, but as it turns out, it cannot be deduced from the adiabatic data alone. In fact, the diabatization is not well-defined because different sets of linear α, β, γ can lead to the same adiabatic PESs. In principle, any one of those sets is equally valid, but when performing this process at different ***q***, we would like to always obtain the same solution. The possible solutions correspond to the different values of the angle ω, so in order to obtain a consistent solution, we must choose ω appropriately.

Let us choose an arbitrary reference structure ***q***^ref^, and define ω(***q***^ref^) = 0. This gives
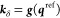
19
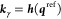
20For any other ***q***, the angle ω can be obtained from
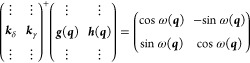
21where ***A***^+^ denotes the Moore–Penrose inverse of ***A***. With this ω, the same linear functions for α, β, and γ will be obtained from any ***q***. Different choices of ***q***^ref^ will result in different diabatic functions, but any of them reproduces the adiabatic data.

To extend the linear model to more general functions, we start by replacing ***k***_*x*_ with ∇*x*(***q***) (*x* ∈ {α, β, γ, δ, τ}). We note that the diabatic-to-adiabatic transformation above still holds, and most of the diabatization would work too, only the selection of ω needs to be modified, because the left-hand side in eq 21 is now unlikely to produce an orthogonal 2 × 2 matrix from which ω can be extracted.

As before, we can select an arbitrary structure as ***q***^ref^ and define the (constant) ***k***_δ_ and ***k***_γ_ with eqs 19 and 20. We realize that in the linear model the ***g*** and ***h*** vectors always span the same plane. In a more general case, the {***g***, ***h***} plane changes with ***q***. So, for any other ***q***, we first transform ***g*** and ***h*** such that they lie in the same plane as ***k***_δ_ and ***k***_γ_. This transformation is based on the singular value decomposition (svd) of the inner product matrix between the two subspaces, and is the “minimal” rotation that achieves it:
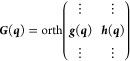
22
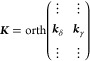
23
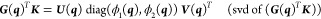
24
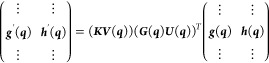
25where orth(***A***) indicates an ortonormalization of the columns of ***A*** by any method and diag(*x*,*y*) a diagonal matrix with diagonal elements {*x*, *y*} . Even though ***g***′ and ***h***′ are now coplanar with ***k***_δ_ and ***k***_γ_, they will probably not correspond to a unitary rotation of the latter, but we can assign a “best fit” value for ω:
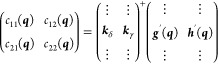
26
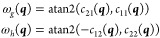
27
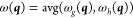
28where avg(*x*, *y*) is the circular mean of two angles, i.e., the angle equidistant and closer to both arguments.

Once a value of ω is defined, the diabatization proceeds as before, that is,
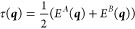
29
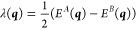
30
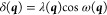
31
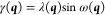
32
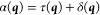
33
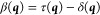
34
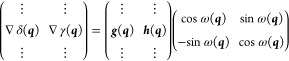
35
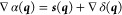
36
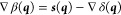
37Note that ***g***′, ***h***′, ***k***_δ_, and ***k***_γ_ are only used to define ω. A last detail is that the ***h*** vector obtained from electronic structure calculations may change sign in an uncontrolled manner, due to the arbitrary phase of the wave functions. To account for this, if the angles ω_*g*_ and ω_*h*_ in eqs 27 and 28 differ by more than π/2, ***h*** is replaced by −***h***.

This achieves a pseudodiabatization (we make no assumption on the size of the couplings between the corresponding states) that we expect to be smooth and consistent at least in the vicinity of ***q***^ref^, even if it contains a CI seam. Farther from ***q***^ref^, especially when the transformation in eq 25 is large (the product ϕ_1_ϕ_2_ is small) and when ω_*g*_ and ω_*h*_ differ significantly, this may not be the case. Moreover, this procedure considers only two surfaces and does not incorporate possible crossings with other surfaces. It is also worth noting that the nature of the wave functions or orbitals involved is never examined, only their energies and gradients/couplings are used.

### 2.3 Surrogate Model

The RVO method is based on a GPR or GEK surrogate model^21−23^ for the PESs.^18^ This surrogate model is built from a set of data (sample) points and exactly reproduces the energies and gradients at the data points—it is an exact interpolator—within the specified tolerance, which is usually set close to machine precision. In particular, the model can be expressed as
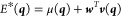
38where μ is the “trend-function” or baseline, ***w*** is a vector of weights, to be optimized when building the model, and ***v*** is a vector of kernel functions and their derivatives. The length of the vectors is the number of independent data used to build the model, i.e., the number of data points (*n*) multiplied by the dimensionality of the PESs (*m*) plus one (all the gradient components and the energy for each data point), *n*(*m* + 1). The kernel function is in general given as *f*(***q***, ***q***′), and the elements of the vector ***v*** at a given ***q*** are the values of *f*(***q***, ***q***_*i*_) and (∇*f*(***q***, ***q***_*i*_))_*k*_ for each data point ***q***_*i*_ and dimension *k*, ordered in some convenient way. The (constant) vector ***w*** is obtained by ensuring that the model reproduces the input data, i.e., that all E*(***q***_*i*_) and ∇E*(***q***_*i*_) match the energies and gradients, respectively, at the data points. This is accomplished by solving the following equation:
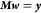
39where ***y*** is a vector that collects the energies (as *E* – μ) and gradients at the data points, in the same order as the ***v*** vector, and ***M*** is the covariance matrix, containing the covariance between the data points, *f*(***q***_*i*_, ***q***_*j*_), as well as their first and second derivatives.

In our case, we use a constant value for the baseline μ, and a Matérn-5/2 covariance function^46,47^ as a kernel function:
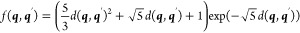
40
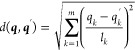
41Here, *d* measures the distance between ***q*** and ***q***′, with each dimension scaled by its *l*-value or “characteristic length”. The *l* values are chosen such that the model built with a single data point reproduces the approximate Hessian matrix given by the HMF^27^ at that point.^18^

Thus, the process to build the pseudodiabatic surrogate model can be summarized as the following:

1. Start with a set of structures, ***q***_*i*_, and the associated *E*^*A*^, *E*^*B*^, ***s***, ***g***, and ***h*** for each structure.
2. Select the latest structure as a reference, ***q***^ref^ = ***q***_*n*_ and set ***k***_δ_ = ***g***(***q***^ref^) and
3. For each structure, obtain the transformed vectors ***g***′ and ***h***′, eqs 22–25, and the angle ω with eqs 26–28. Possibly flip the direction of ***h***.
4. For each structure, obtain α, β, γ, ∇α, ∇β, and ∇γ, using eqs 29–37.
5. Build three independent GEK surfaces, eq 38, from {α(***q***_*i*_), ∇α(***q***_*i*_)}, {β(***q***_*i*_), ∇β(***q***_*i*_)}, and {γ(***q***_*i*_), ∇γ(***q***_*i*_)}.

The adiabatic energies and gradients can be obtained from these surfaces by diagonalizing the corresponding Hamiltonian, eq 6, and they reproduce by construction the initial data in step 1, except for the possible sign flip of ***h***.

### 2.4 Optimization

The RVO method, based on a GEK surrogate model, has been described in previous works.^18−20^ In short, the surrogate model is built from the electronic structure data of the previous iterations, and a stationary point is then located on the surrogate model through a number of microiterations. The progress of the microiterations is limited by the uncertainty or predicted variance of the surrogate model, which, for the case of GEK, can be computed as
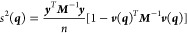
42such that the 95% confidence interval for the prediction is . Once the stationary point is found on the surrogate model (or the maximum variance is encountered), a new electronic structure calculation is performed for that geometry and that completes a macroiteration. For the next macroiteration, a new surrogate model is built, now including the data just computed.

Constraints of different types can be included in the optimization thanks to the PCO.^19,35^ This method is based on defining a unitary transformation of the coordinates ***q*** that allows separating these degrees of freedom into two subspaces, one that is constrained and one that is optimized. At each microiteration, the coordinates in the constrained subspace are modified in order to fulfill the constraints, while the coordinates in the optimized subspace are modified with a general optimization method such as, for example, RS-RFO.

In ref (19) it was noted that the implementation at the time did not support the use of nongeometrical constraints with RVO because it needs the possibility of obtaining the value of the property being constrained during the microiterations when no electronic structure calculations are performed. The optimization of MECPs is one of the cases that involves nongeometrical constraints,^31,36^ in particular the energy difference between two states is constrained to zero. Specifically, the optimization of CIs requires not only the energy difference between two states but also the nonadiabatic coupling vector between them. With the pseudodiabatization described above, all the required quantities can be obtained from the surrogate model, and the PCO can be applied as with any other constraint.

The case of crossings between states of different spin multiplicity is similar to CIs, but the process is somewhat simplified. When there is no coupling between the states, it can be assumed that γ is identically zero everywhere and therefore *E*^*A*^ = α, *E*^*B*^ = β, only two surfaces are needed (***h*** is not used either), and no pseudodiabatization is required as there is no singularity.

## 3 Computational Details

The methods described above have been implemented in OpenMolcas^48,49^ and are publicly available in its latest version. This software has been used for all the quantum chemistry calculations in this work. As in previous works,^18,19^ we set a baseline value μ for the GEK surrogate models that is 10.0*E*_h_ above the maximum energy value among the data points. This is done independently for each of the energy surfaces (α, β), but for the γ surface we set μ = 0. The *l* values obtained from the HMF are used for all the surfaces.

**Figure 2:**
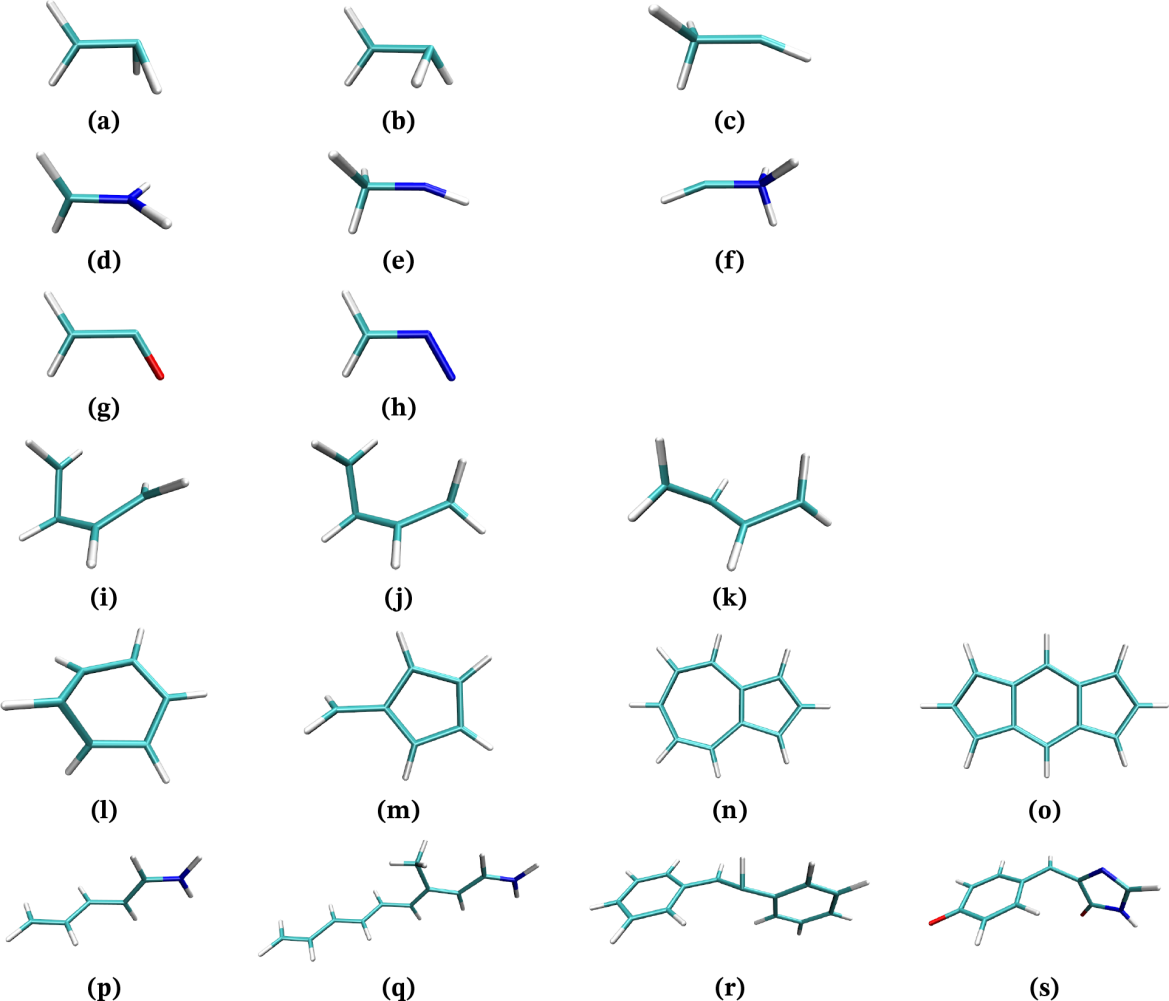
Structures studied in this work, shown at their optimized S_0_/S_1_ MECP from ref (36).

The optimization of MECPs has been tested for the same systems as in ref (36) (Figure 2), with similar settings. Optimizations were done at the state-average complete active space self-consistent field (SA-CASSCF) level, the basis set was ANO-RCC with double-ζ-plus-polarization contraction,^50^ and the atomic compact Cholesky decomposition (acCD)^51^ was employed in all calculations to treat two-electron integrals, with the default threshold of 10^–4^*E*_h_. The convergence thresholds for the optimizations were the defaults in OpenMolcas (rms displacement and step size of 1.2·10^–3^*a*_0_ and 3.0·10^–4^*E*_h_*a*_0_^–1^, respectively; maximum components 1.5 times these values), plus a requirement for the energy difference between the crossing states to be below 10^–5^*E*_h_. No spacial symmetry was enforced in any case.

For each system, at least one S_0_/S_1_ MECP was optimized; the number of active electrons, orbitals, and averaged states for each case is specified in Table1. The starting structures are the same as in ref (36).

**Table 1:** Active Space Specifications for the S_0_/S_1_ MECP Optimizations^a^

Molecule	Structure	(*n*_e_,*n*_0_)	*n*_s_
ethylene	**(a)**, **(b)**, **(c)**	(2,2)	2
methaniminium	**(d)**, **(e)**, **(f)**	(2,2)	2
ketene	**(g)**	(2,3)	2
diazomethane	**(h)**	(2,3)	2
butadiene	**(i)**	(4,4)	3
”	**(j)**, **(k)**	(4,4)	2
benzene	**(l)**	(6,6)	2
fulvene	**(m)**	(6,6)	2
azulene	**(n)**	(10,10)	2
*s*-indacene	**(o)**	(12,12)	2
PSB3	**(p)**	(6,6)	2
Me-PSB5	**(q)**	(10,10)	2
stilbene	**(r)**	(2,2)	3
GFP chromophore	**(s)**	(2,2)	3

a *n*_e_, *n*_o_, *n*_s_: number of electrons, orbitals and states, respectively, in the SA-CASSCF procedure.

Additionally, from the same starting structures we optimized S_0_/T_1_ MECPs. For most of these calculations, the same active spaces as those in Table 1 were used, but with no state averaging, as both singlet and triplet states are the lowest in their multiplicity. The differences and exceptions are listed in Table 2; in particular, for **(n)** and **(o)**, the S_1_/T_1_ MECP was optimized instead, as the proximity of the S_0_/S_1_ crossing made the optimization unstable.

**Table 2:** Specific Details for the S_0_/T_1_ MECP Optimizations^a^

Structure	Specific changes
**(g)**, **(h)**	S_0_ with SA(2)
**(i)**, **(j)**, **(k)**	CASSCF(2,2)
**(m)**	S_0_ with SA(2)
**(n)**, **(o)**	S_1_/T_1_ MECP, S_1_ with SA(2)
**(r)**, **(s)**	S_0_ with SA(3)

a By default, active spaces are the same as those in Table 1, with no state averaging. The notations CASSCF(*n*_e_,*n*_o_) and SA(*n*_s_) are used.

Root mean square deviations (rmsd) between molecular structures were computed with the rmsd Python package,^52^ considering possible mirrorings and atom permutations to minimize the difference.

## 4 Results

We show first the results for the S_0_/T_1_ optimizations. Table 3 compares the optimizations performed with the conventional RS-RFO method and with the RVO as newly implemented for MECPs. Apart from the number of iterations needed to reach convergence, the rmsd between the optimized structures of both methods is also given, as well as the difference between the optimized MECP energies (*E*^×^), where a negative sign indicates the RVO structure is more stable.

**Table 3:** Number of (Macro)iterations to Converge the S_0_/T_1_ MECP structures, rmsd and Energy Difference between the Two Methods (Δ*E*^×^ = *E*_RVO_^×^ – *E*_RS–RFO_^×^)

	RS-RFO	RVO	rmsd (pm)	*ΔE*^×^ (m*E*_h_)
**(a)**	24	22	0.034	–0.0001
**(b)**	10	14	0.055	–0.0002
**(c)**	10	10	2.267	–0.0167
**(d)**	6	6	0.007	–0.0000
**(e)**	10	7	0.008	0.0003
**(f)**	34	10	0.989	–0.0034
**(g)**	8	6	0.005	0.0005
**(h)**	9	7	0.011	0.0000
**(i)**	17	13	0.019	0.0000
**(j)**	16	15	0.007	0.0000
**(k)**	14	11	0.025	–0.0001
**(l)**	14	12	0.019	0.0000
**(m)**	12	11	0.142	–0.0018
**(n)**^a^	6	6	0.003	–0.0007
**(o)**^a^	6	6	0.023	–0.0002
**(p)**	8	10	0.044	–0.0001
**(q)**	30	31	0.059	–0.0000
**(r)**	26	14	0.038	0.0001
**(s)**	35	18	0.152	–0.0003

a S_1_/T_1_ MECP.

The first thing to notice is that in most cases RVO converges in fewer iterations than RS-RFO. Even in some cases where RS-RFO is efficient, RVO can still save one or two iterations, and in more difficult cases, like **(r)** and **(s)**, the savings can be more significant. In general, the differences in both geometry and energy are very small, indicating that the two methods converged to essentially the same structure. The cases where the results seem to be significantly different are **(c)**, **(f)**, **(m)**, and **(s)**, and in these, not only does RVO take fewer (or as many) iterations than RS-RFO but it also achieves a lower final energy.

The optimized S_0_/T_1_ MECP for **(c)** is characterized by a H–C–C–H dihedral close to 180°. It is 179.3° with RVO but 175.2° with RS-RFO. Similarly, in the case of **(f)** the H–N–C–H dihedral is 179.2° with RVO and 177.4° with RS-RFO. The differences in **(m)** and **(s)** are much smaller and not worth detailing.

It should be noted that the structures in Figure 2 are S_0_/S_1_ MECPs, so they do not reflect in all cases the structure of the S_0_/T_1_ MECP. For example, **(a)** and **(b)** converge to the same twisted structure, similar to **(d)**, while **(i)**, **(j)**, and **(k)** converge to a structure with a 3-member ring, and **(m)** is almost planar.

Overall, it seems clear that at least for these systems the RVO method represents an improvement over the conventional RS-RFO. We would like to point out that although the RVO optimization is computationally more expensive than RS-RFO, this cost increase is completely negligible compared to the cost of the electronic structure calculations, and the number of iterations is therefore an accurate measure of performance, at least for systems of up to a few dozen atoms.

Having established the good behavior of RVO with two surfaces, we discuss now the results for S_0_/S_1_ MECP optimizations, where the surrogate model is given by the pseudodiabatic surfaces α, β, and γ. The comparison between RVO and RS-RFO is given in Table 4. The difference now is more important than for the S_0_/T_1_ MECPs. Only for **(e)** and **(p)** does RVO take one or two more iterations (and it still converges to lower energy), while in all other cases it takes significantly fewer iterations, sometimes less than half. In terms of rmsd and energy differences, **(a)** and **(e)** stand out, while **(m)**, **(p)**, and **(r)** are also slightly larger than the rest.

**Table 4:** Number of (Macro)iterations to Converge the S_0_/S_1_ MECP Structures, rmsd and Energy Difference between the Two Methods (*ΔE*^×^ = *E*_RVO_^×^ – *E*_RS-RFO_^×^)

	RS-RFO	RVO	rmsd (pm)	Δ*E*^×^ (m*E*_h_)
**(a)**	34	5	21.065	5.3841
**(b)**	14	7	0.097	–0.0011
**(c)**	20	13	0.031	0.0003
**(d)**	5	4	0.007	–0.0005
**(e)**	14	15	8.417	–0.9213
**(f)**	16	14	0.024	0.0008
**(g)**	10	7	0.010	–0.0005
**(h)**	10	7	0.004	–0.0004
**(i)**	19	10	0.027	–0.0002
**(j)**	29	11	0.017	0.0000
**(k)**	12	10	0.023	–0.0001
**(l)**	7	6	0.021	0.0003
**(m)**	17	13	0.349	–0.0008
**(n)**	9	6	0.016	0.0025
**(o)**	6	5	0.009	–0.0002
**(p)**	8	10	0.123	–0.0002
**(q)**	32	23	0.025	0.0001
**(r)**	34	17	0.119	–0.0005
**(s)**	17	11	0.055	0.0006

In the case of **(a)**, RVO converged to the symmetric structure shown in Figure 2, but RS-RFO found the same as **(b)** instead. As discussed in ref (36), the symmetric structure is not a minimum but rather a saddle point in the intersection space, and with RS-RFO, probably due to numerical noise, the symmetry is broken and the optimization falls to a minimum, which explains the large number of iterations and lower energy found with RS-RFO. For **(e)**, the main structural difference is the C–N–H angle, which is 159.6° with RVO and 174.0° with RS-RFO. Given the rather large energy difference between both structures, it does not look like the surface is very flat, and we assume that in this case RS-RFO got stuck at or close to a saddle point.

**Figure 3:**
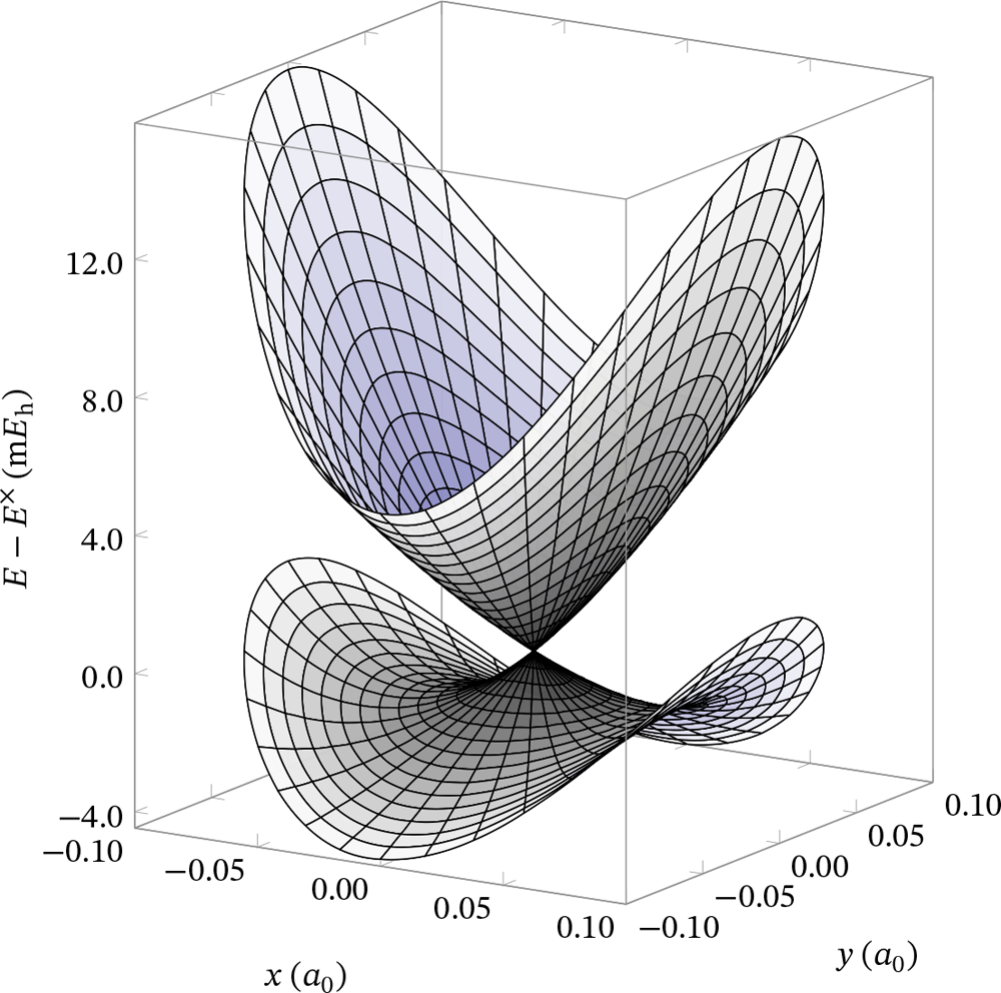
Representation of the adiabatic PESs obtained with the GEK surrogate model around the optimized S_0_/S_1_ MECP of **(p)**.

**Figure 4:**
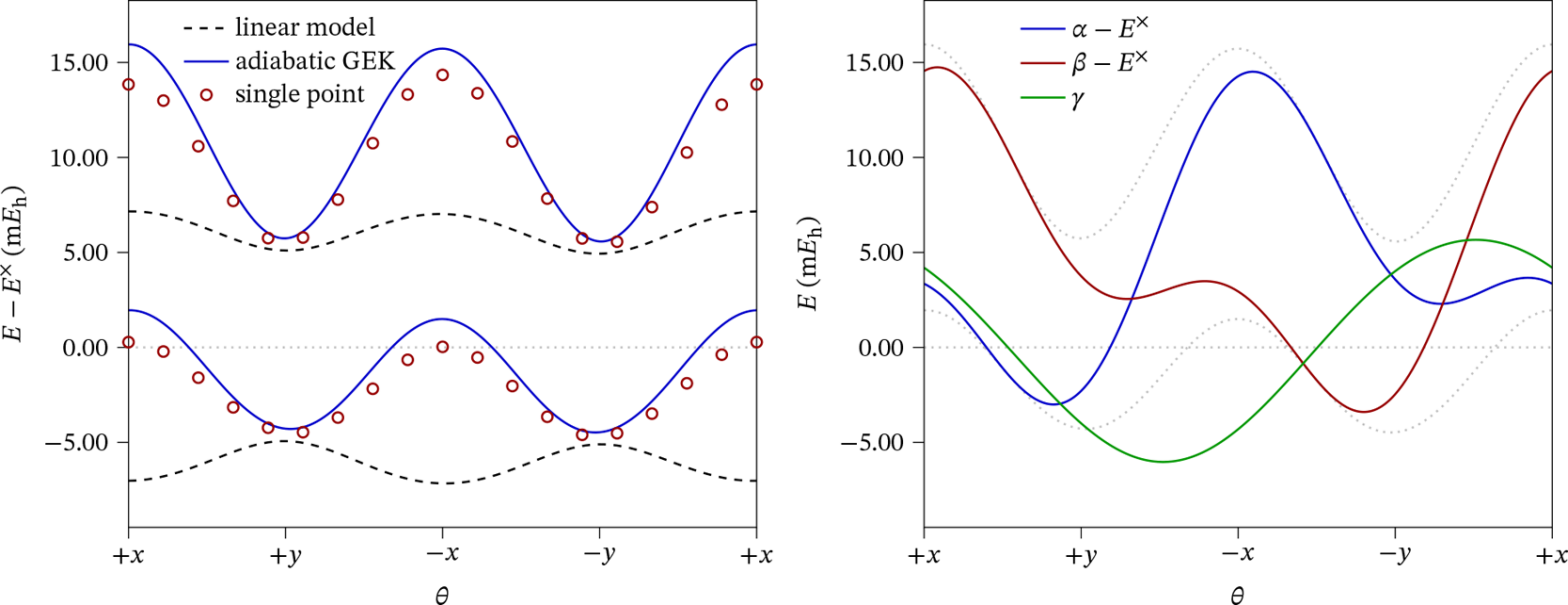
Plot of the PESs in a circle around the optimized S_0_/S_1_ MECP of **(p)** with a radius of *r* = 0.1 *a*_0_, in the branching plane. Left: adiabatic model surfaces obtained with the linear model and with the GEK surrogate model, as well as single-point calculations at selected geometries. Right: The three pseudodiabatic surfaces from which the adiabatic GEK on the left (shown also as dotted curves) is obtained.

Although the surrogate model built for RVO is only intended to be used for optimization purposes (not, for instance, to run molecular dynamics simulations on it), it is instructive to examine how well it reproduces the “true” surfaces around a CI and how it differs from a simpler linear model. We take as an example the optimized S_0_/S_1_ MECP of **(p)**. In ref (36), the linear model was already analyzed for this system, and it was found that while it was valid for regions very close to the CI, it deviates appreciably from the computed energies farther away, and can give qualitatively wrong predictions beyond ∼0.03 *a*_0_. We represent in Figure 3 the shape of the adiabatic surfaces obtained from the GEK surrogate model in the branching plane around the MECP; up to a distance of 0.1 *a*_0_, the deviation from the linear model is evidenced by the curved shape of the radial grid lines, particularly clear in the lower surface. We then compare this GEK prediction with actual SA-CASSCF single-point calculations for structures around the rim of this figure and plot them in Figure 4. It is seen that the linear model (wrongly) predicts minima along the ±*x* direction; the single-point calculations, however, show that the real energies are much higher. The model obtained from the GEK surfaces follows much more closely the computed energies, although there are still some deviations. It must be emphasized that the GEK model is not built to reproduce these energies, but only those of the latest 10 iterations between the initial structure and the final optimized MECP. The right panel of Figure 4 shows the corresponding α, β, and γ surfaces. Similar comparisons for the other systems confirm that the GEK model provides a much better approximation to the SA-CASSCF energies than a simple linear model.

In both S_0_/S_1_ and S_0_/T_1_ MECP optimizations, it was found that the structure that needed most iterations to converge was **(q)**. This correlates to its being the most flexible system in the set, but we observe that in this case most of the iterations, for the two optimization methods, are spent in a 60° rotation of the CH_3_ group, which results in a stabilization of around 1.1 kcal mol^–1^. It can be expected that an overshooting procedure such as the one implemented in ref (24) could improve the performance of RVO, especially when the surrogate model is expressed in internal coordinates. However, we did not use overshooting, so this remains a possible area of improvement.

As a summary, for S_0_/T_1_ MECPs, the use RVO reduced the total number of iterations from 295 to 229 (a 22% reduction), for S_0_/S_1_ MECPs the reduction is from 279 to 189 (32%), excluding **(a)**, where both methods converge to clearly different structures.

## 5 Conclusions

We have implemented a pseudodiabatization process that allows representing the crossing between two adiabatic PESs as a combination of three smooth, continuous pseudodiabatic surfaces. This is used to build a surrogate model for the RVO method in order to efficiently locate MECPs. The test calculations reported here indicate that this extension to RVO achieves a noticeable reduction in the number of iterations (energy and gradient evaluations) required, both for crossings between states of different spin and for CIs. It can be noted that although most of the test MECPs in this work involve the S_0_ ground state, there is nothing in the method that is specific for the ground state, so it can be straightforwardly applied to crossings between excited states.

The properties used to build the surrogate model are only those used in the conventional optimization: energies, gradients, and nonadiabatic coupling. A comparison of the model with single-point energy calculations shows that the adiabatic PESs around a CI are well approximated beyond what a linear model can provide. However, it is worth a reminder that the model is only intended to be a local approximation in the vicinity of the final optimized structure and not as a global representation of the surfaces.

## Supporting Information Available

Input files and relevant output files for each of the MECP optimizations with OpenMolcas (ZIP)
